# Smooth pursuit operates over perceived not physical positions of the double-drift stimulus

**DOI:** 10.1167/jov.21.11.6

**Published:** 2021-10-08

**Authors:** Marvin R. Maechler, Nathan H. Heller, Matteo Lisi, Patrick Cavanagh, Peter U. Tse

**Affiliations:** 1 Department of Psychological and Brain Sciences, Dartmouth College, Hanover, NH, USA; 2 Department of Psychology, University of Essex, Colchester, UK; 3 Department of Psychology, Glendon College and CVR, York University, Toronto, Ontario, Canada

**Keywords:** illusion, smooth pursuit, motion perception

## Abstract

The double-drift illusion produces a large deviation in perceived direction that strongly dissociates physical position from perceived position. Surprisingly, saccades do not seem to be affected by the illusion (Lisi & Cavanagh, 2015). When targeting a double-drift stimulus, the saccade system is driven by retinal rather than perceived position. Here, using paired double-drift targets, we test whether the smooth pursuit system is driven by perceived or physical position. Participants (*n* = 7) smoothly pursued the inferred midpoint (Steinbach, 1976) between two horizontally aligned Gabor patches that were separated by 20° and moving on parallel, oblique paths. On the first half of each trial, the Gabors’ internal textures were static while both drifted obliquely downward. On the second half of each trial, while the envelope moved obliquely upward, the internal texture drifted orthogonally to the envelope's motion, producing a large perceived deviation from the downward path even though the upward and downward trajectories always followed the same physical path but in opposite directions. We find that smooth pursuit eye movements accurately followed the nonillusory downward path of the midpoint between the two Gabors, but then followed the illusory rather than the physical trajectory on the upward return. Thus, virtual targets for smooth pursuit are derived from perceived rather than retinal coordinates.

## Introduction

**Figure 1.**
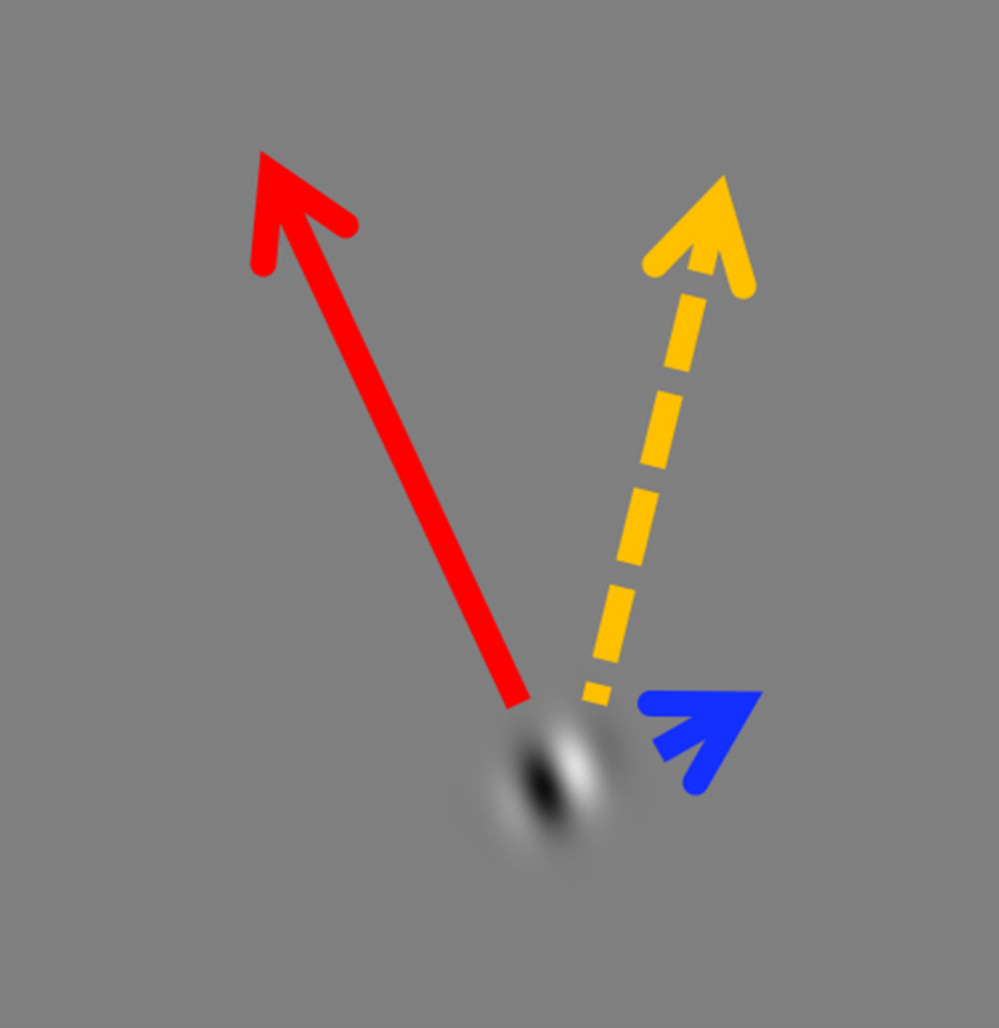
When viewed peripherally, a Gabor patch moving over a gray background will appear to travel in the direction (yellow arrow) that is a combination of its external direction (red arrow) and the direction of its internal grating (blue arrow).

Motion-induced position shifts are a class of illusion where the presence of motion causes the misperception of position (Cavanagh & Anstis, 2013; Duncker, 1929; Eagleman & Sejnowski, 2007; Wallach et al., 1978; Whitney, 2002). These illusions have been used to investigate ways in which different sources of low-level stimulus information are combined in the visual system before the formation of a conscious representation. A particularly striking example of this class of illusion is the double-drift stimulus (Lisi & Cavanagh, 2015), which is also known as the curveball illusion (Kwon et al., 2015; Shapiro et al., 2010) and originally as the infinite regress illusion (Tse & Hsieh, 2006). The double-drift stimulus contains two sources of motion information: an envelope that translates across the screen (i.e., the external drift) and a moving visual texture that is confined within the envelope (i.e., the internal drift). When these two sources of motion are oriented orthogonally, the visual system combines them to produce an intermediate motion percept. This process results in a dramatic misperception of both the position of the stimulus and the direction of its motion when viewed peripherally (Figure 1 and Movie S1). The dissociation between an object's physical position (i.e., where it is encoded on the retina) and its perceptual position (i.e., where it is represented in consciousness) provides a probe that can be used to determine whether physical or perceived position drives downstream cognitive operations and motor actions.

**Figure 2.**
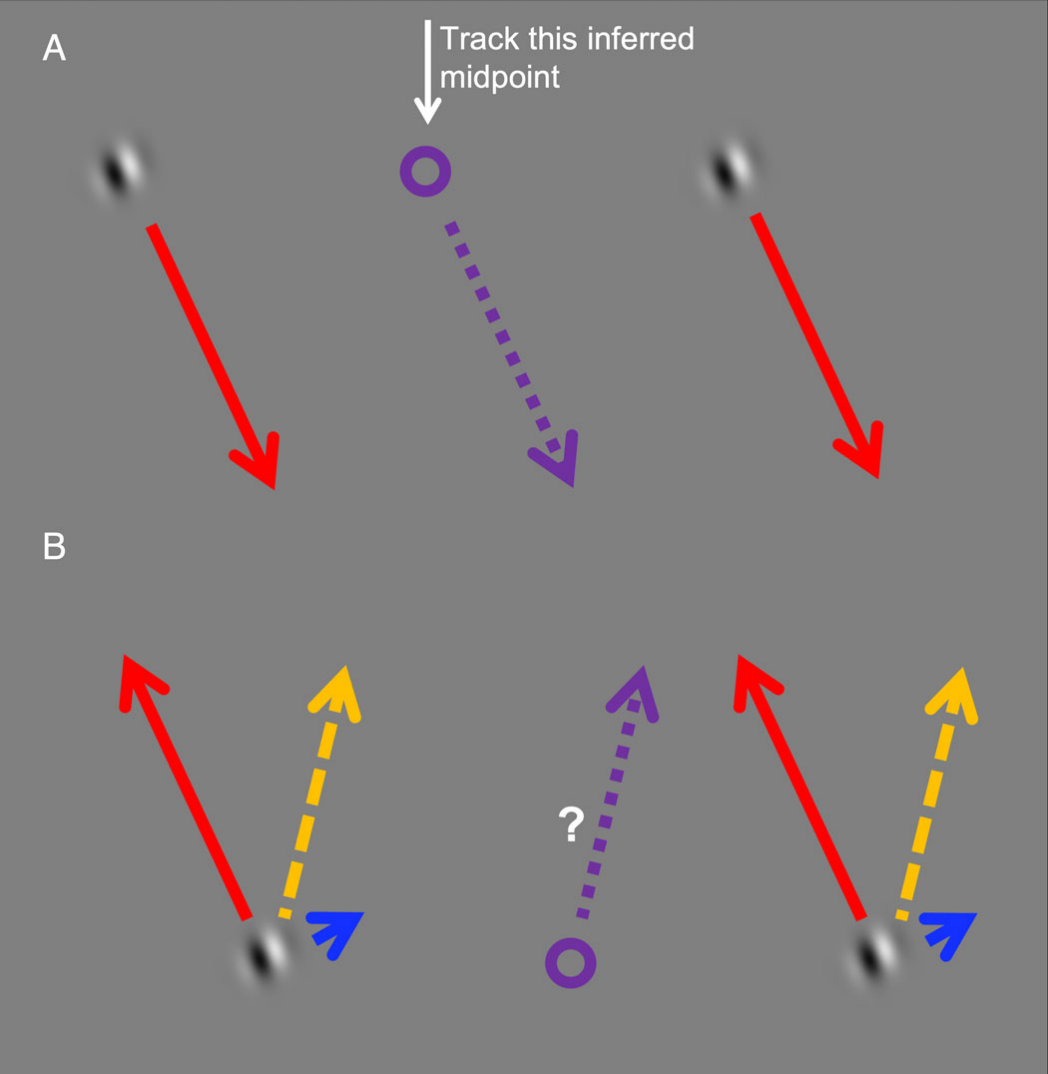
Schematic of the stimulus. (A) During the first part of the trial the physical path (red arrows) of both Gabors is downward and the fixated midpoint (purple circle) follows the same trajectory (purple dotted arrow). (B) When the Gabors begin to return upward along the same physical path, the orthogonal internal motion (small blue arrow) creates an illusorily perceived trajectory (yellow arrow). The fixated midpoint follows this illusorily perceived trajectory. See Movie 1 for a demonstration.

Using the double-drift stimulus as a probe, Lisi and Cavanagh (2015) found that saccades were made not to the probe's perceived location, but rather to its physical location. This is a dramatic example of an action process that is uncoupled from conscious experience (Goodale & Milner, 1992). Subsequent results have shown that this dissociation between action and consciousness may be unique to saccades that are programmed while the stimulus is present on the screen. For example, when the stimulus is removed from the screen and the saccade system must target a memory representation of the stimulus instead, saccades are driven toward the perceived position (Massendari, Lisi, Collins, & Cavanagh, 2018; Ueda, Abekawa, & Gomi, 2018), suggesting that memories are stored in a perceptual format. Likewise, motor actions involving hand motion, such as pointing movements and tracking with a stylus, are guided by the perceived position and not the physical position (‘t Hart, Henriques, & Cavanagh, 2019; Lisi & Cavanagh, 2017). Moreover, it has been shown that a number of higher order perceptual and cognitive operations, including attentional tracking (Maechler, Cavanagh, & Tse, 2021), pop-out effects (Özkan, Tse, & Cavanagh, 2020), and the computation of apparent motion trajectories (Hui et al., 2020), are all driven by the perceptual representation of the stimulus.

In this study, we investigate a second eye movement system that can act while the stimulus is present—smooth pursuit—and ask whether it is driven by the physical or the perceived position of the double-drift probe. It was recently shown that the illusion itself survives smooth pursuit. Cavanagh and Tse (2019) had participants pursue a smoothly moving fixation point while the Gabor patch, located 18.75° of visual angle away from fixation, followed the same trajectory as the fixation point. In this way, the envelope was approximately stabilized on the retina during pursuit. This condition nulls retinal sources of motion information about the envelope's trajectory, leaving primarily information generated by the pursuit system. Cavanagh and Tse (2019) found that the illusion persists without loss during smooth pursuit. Thus, efference copy from the pursuit system is combined with local motion information generated by the texture (i.e., the internal drift) to produce offsets in both position and direction. Therefore, the illusion must be computed after the recovery of motions in the world based on eye movement signals.

In their experiment, Cavanagh and Tse (2019) had participants smoothly pursue a high contrast fixation spot while the double drift patch was in the periphery. Here we test whether smooth pursuit of a virtual target derived from an illusory stimulus is affected by the illusion. To do so, we adapted a paradigm in which participants pursued the inferred midpoint between two objects (Beutter & Stone, 2000; Hafed & Krauzlis, 2008; Steinbach, 1976). Two Gabors moved in tandem down and then up the screen, while participants tracked the virtual midpoint halfway between the two Gabors. On the way down, the Gabors had no internal drift, but on the way up the internal grating drifted orthogonally to the external motion direction, creating the classic double-drift effect. If smooth pursuit is driven by physical position as is the case for saccades, the gaze trajectories would have the same angle in both downward and upward segments, because the Gabors in fact traversed the same path on the way down as on the way up. However, if perception determines smooth pursuit targets, then the trajectories and their angles should diverge, because the double-drift illusion was only present during the upward trajectory.

Many other studies have investigated whether smooth pursuit follows the physical or perceived path of a stimulus (see Spering & Montagnini, 2011, for a review). Overall, the existing literature is split on the issue. For instance, two previous studies have looked at whether smooth pursuit is based on retinal or perceived target trajectories using the Duncker illusion to make the perceived path deviate from the physical path (Wyatt & Pola, 1979; Zivotofsky, 2005). The two articles came to opposite conclusions. Previous research (Hughes, 2018; Zhang, Yeh, & DeValois, 1993) has revealed that a Gabor patch seems to move faster if both internal and external motions have the same direction and slower if the two motions have opposite directions. A study examining smooth pursuit of Gabor patterns with internal motion in the same or opposite direction as the envelope motion found that the pursuit gains were influenced by the drift in the same way as perception (Hughes, 2018). However, in the same experiment the perceptual error and the pursuit error were not correlated, suggesting a dissociation of perception and action in smooth pursuit. In contrast, two studies reported smooth pursuit of a static stimulus that seemed to move because of motion aftereffects, linking perception and smooth pursuit (Braun, Praceus, & Gegenfurtner, 2006; Matsumiya & Shioiri, 2015). Other investigations into conflicts between action and perception using visual illusions have also found mixed results (Bruno, 2001). Here, the smooth pursuit that we observed for a virtual target derived from an illusory stimulus showed a strong effect of the illusion on eye movement trajectories.

## Methods

### Participants

We recruited seven people as participants (six men and one woman, mean age 32 years, six right handed), two of whom are authors of this article. The experiment was approved by the Institutional Review Board at Dartmouth to ensure compliance with ethical standards. Additionally, all procedures were approved by Dartmouth's Environmental Health and Safety department regarding COVID-safe experimentation.

### Stimuli

Two Gabor patches—sinusoidal luminance gratings with a Gaussian envelope—served as stimuli for this experiment. The visible diameter of a Gabor was approximately 0.5° of visual angle (dva), with a sigma of 0.1 dva and spatial frequency of 2 cycles per degree. They were aligned on a horizontal line and separated by 20 dva (10 dva in each direction from their midpoint). At the beginning of each trial, there was also a black fixation spot with 0.2 dva diameter in the middle between the two Gabors, which disappeared once the trial started.

Participants initiated a trial by fixating their gaze on the fixation spot for half of a second. After the fixation spot disappeared, the two Gabors, neither of which had internal motion, started moving downward toward either the left or right lower corner of the screen along parallel paths, at an angle of ±22° relative to vertical. Participants were asked to fixate and smoothly pursue the inferred (but invisible) midpoint between the two Gabors. To facilitate smooth pursuit eye movements and diminish the need to make catch-up saccades, the Gabors accelerated and decelerated in a sinusoidal fashion over the whole path. After traversing a 2 dva long path within 1 second (i.e., their average movement speed was 2 dva/s), the Gabors reversed direction and moved back along the same path at the same speed. On the way back, however, their internal grating continuously drifted at 2 dva/s. The drift direction of the internal grating was orthogonal to the Gabors' external motion paths, creating the double-drift stimulus. Although the Gabors moved up the screen along the same path that they traversed on the way down, they looked as if they were following a V-shaped trajectory. See figure 2 as well as Movie S1 in the supplementary material for a demonstration of the stimulus.

### Procedure

Participants initiated the motion of the Gabors by fixating the black dot between the Gabors, which disappeared after fixating for half of a second. They were then tasked with keeping their gaze fixated on the moving, invisible midpoint between the two Gabors. If smooth pursuit follows the image of an object on the retina, there should be no difference between pursuing drifting or nondrifting Gabors. However, if smooth pursuit follows the perceived position of an object, then the added internal drift should make the participants’ gaze deviate from the veridical midpoint of the Gabors (Figure 2). Using an eye tracker (EyeLink 1000, SR Research, Oakland ON, Canada; 1000 Hz sampling rate) we recorded and monitored participants’ gaze during the experiment. When their gaze deviated from the Gabors’ midpoint along the *y*-axis by more than 2 dva, the trial was repeated. Because we expected deviations along the *x*-axis to be induced by our stimulus, trials were not restarted when the gaze deviated along the *x*-axis. We ran a total of 200 trials per participant, counterbalanced and pseudorandomized for the initial motion direction (tilted left/right). The experiment was self-paced and took approximately 15 to 20 minutes to complete.

There was a small limitation with regard to the calibration of the eye tracking equipment, which could not be done by the experimenter owing to restrictions related to the COVID-19 pandemic. Instead, participants had to calibrate the eye tracker and other equipment using oral instructions from the experimenter, who was monitoring the experiment from an adjacent room. This factor might have had a small impact on the overall accuracy of the eye tracking, but because all comparisons were done within each trial, the resulting noise in the data was well-controlled.

### Apparatus

Participants viewed the stimuli on an AM250 OLED monitor (Flanders Scientific Inc, GA; Cooper et al., 2013). The screen was set to 1920 × 1080 resolution. We used the Psychophysics Toolbox (Brainard, 1997; Pelli, 1997) for the creation and display of the stimuli. Eye movements were monitored live and recorded using an EyeLink 1000 eye tracker (SR Research, Oakland ON, Canada; 1000 Hz sampling rate). Participants were stabilized with a chin rest and a forehead rest at a distance of 58 cm from the screen.

## Results

**Figure 3.**
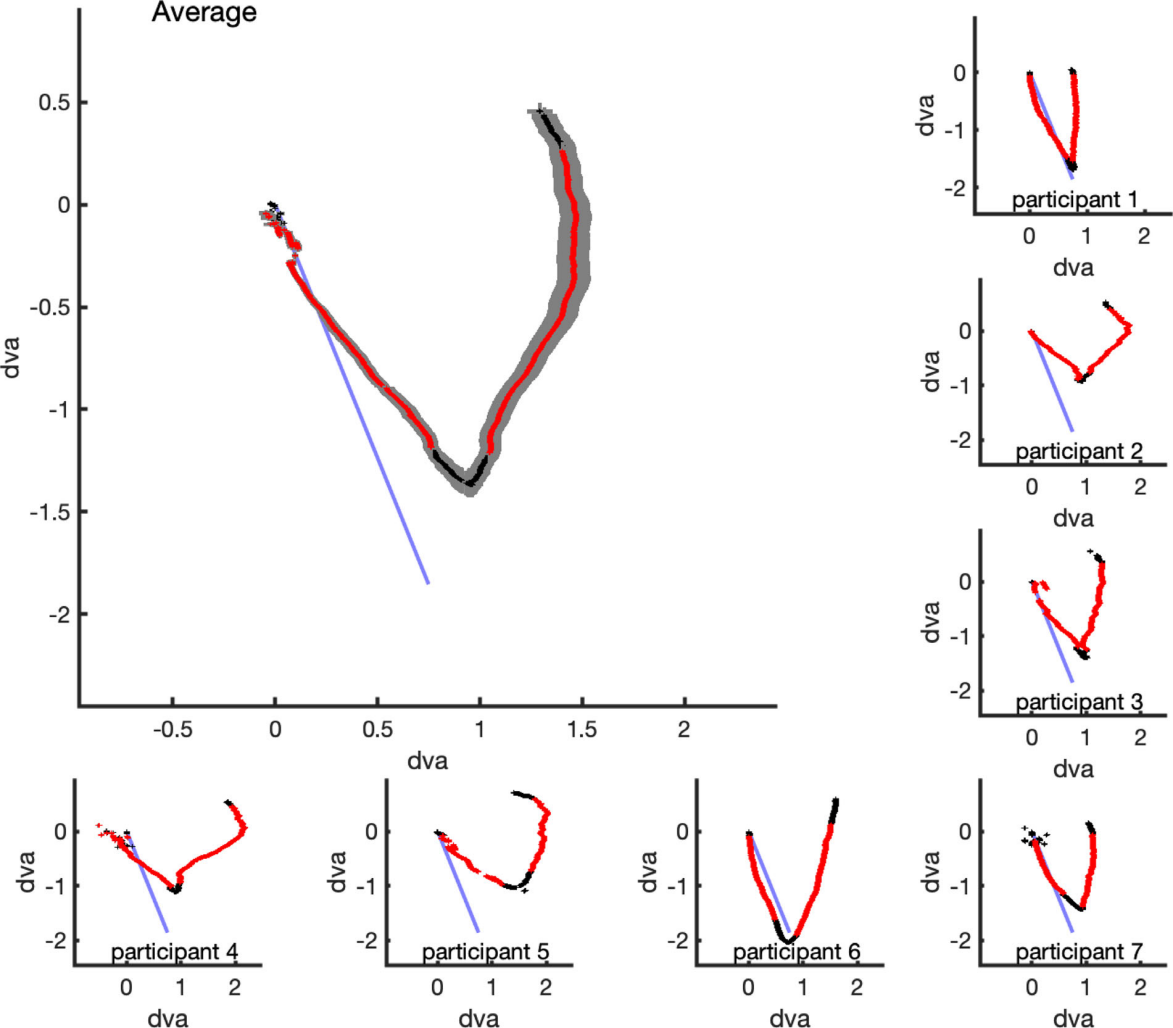
Average individual and group eye movement traces. Blue lines represent the path of the midpoint between the two Gabors on the screen (i.e., where participants were told to look). The black and red traces correspond with the mean trajectory of gaze starting at the origin (i.e., where they actually looked). The shaded area in the group average represents the standard error of the mean. Red segments of the traces were used in the analysis of the angles. Areas where the line seems to be broken coincide with time points when many saccades caused outliers in recorded gaze positions (i.e., beginning, end, and reversal). The black 200-ms segments of the traces were excluded from the data analysis.

**Figure 4.**
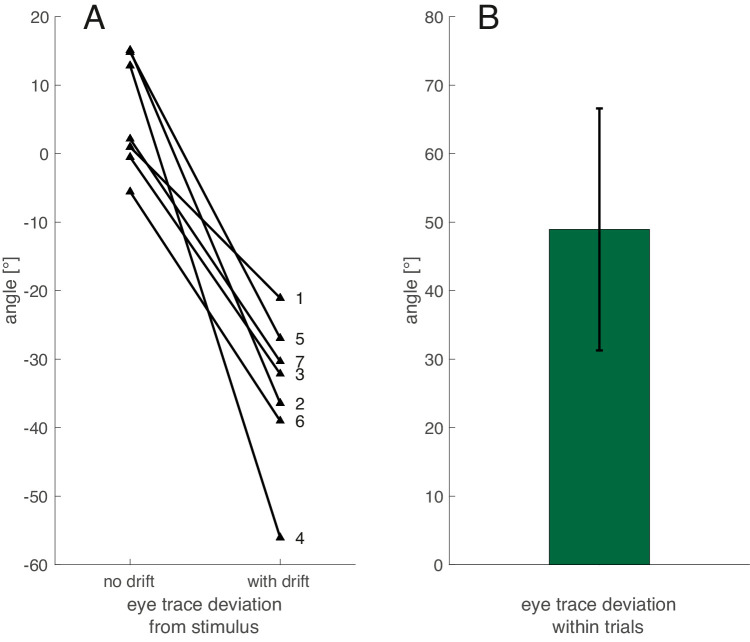
Differences in direction of eye traces with and without internal drift. (A) Deviations in gaze tilt from the actual midpoint of the two Gabors. (B) Average of within trial difference between the two traces and the 95% confidence interval. Numbers correspond with the individual participants as in Figure 3.

As a first analysis step, the eye movement trace from each trial was split into two halves: the initial segment without internal drift of the Gabors and the second segment with internal drift. Next, we trimmed each half by 200 ms worth of gaze data at the beginning and end to exclude eye movement instabilities associated with starting, ending, and reversal, such as catch-up saccades. Figure 3 shows individual eye movement traces including saccades. We then used orthogonal regression (Deming, 1943) to fit two lines, one to each central portion of the downward and upward traces. The initial, downward segment without the illusory stimulus showed a good match between the path of the virtual midpoint and the eye movement path with an average deviation of 5.70° (95% confidence interval, −2.07° to 13.46°). The angle fit to the second segments varied widely across participants (ranging from −26.34° to −74.02°), but deviated consistently from the physical path in the same direction for all participants (see Figure 4A). The average deviation was −34.57° (95% confidence interval, −44.90° to −24.24°). We tested the difference between the angles of the first and second segments as a measure of the influence of the illusion on smooth pursuit. The average difference was 48.94°, significantly different from zero, 95% confidence interval, 31.28°–66.61°, *t*(6) = 6.78, *p* < 0.001, Cohen's *D* = 2.56, 62.1% explained variance (see Figure 4B). When the analysis was repeated without excluding the initial start-up, reversal and end segments (black portions of the average gaze trajectory on Figure 3), the results were essentially the same.

Our first analysis revealed that the direction of the eye movements depended on perception, but it is possible that this effect was driven by saccades to the perceived midpoint, rather than by actual smooth pursuit of the perceived midpoint. To address this concern, we conducted multiple control analyses. First, with the goal of analyzing only trials without saccades, we used the algorithm of Kliegel and Engbert (2003) to detect saccades and microsaccades. This strategy allowed us to remove all trials from the analysis in which a saccade occurred during the double-drift part of the trial (66.9% of all trials). The downward segment of each trial still overlapped well with the physical midpoints (average angle between physical path and eye trace: 2.59°, 95% confidence interval, −5.81° to 11.00°), and in the double-drift condition the angle between eye trace and stimulus was still significantly larger (average 31.1°; 95% confidence interval, 27.54°–34.66°). The average within-trial difference in the eye trace angle (33.69°) was also significantly greater than zero, 95% confidence interval, 26.88°–40.5°, *t*(5) = 12.72, *p* < .001, Cohen's *d* = 5.19, 87.1% of variance explained, albeit smaller than when we included all trials.

We also calculated eye velocity gain for each half of each trial. Instead of removing entire trials with saccades, we de-saccaded the pursuit traces by removing segments that were identified as saccades. Then we calculated the average speed of the eye movements separately in each half of each trial. Because the illusion moves the perceived position of the Gabors away from the physical position along the *x*-axis, but not the *y*-axis, this analysis is matched for both conditions (with and without internal drift). The mean gain along the *y*-coordinate while the Gabors drifted downwards (without illusion/internal drift) was 0.6. Comparing this finding with the mean gain along the *y*-axis while the Gabors drifted upward (with internal drift/illusion) showed no significant difference; the mean gain in this condition was 0.73, *t*-test for difference between the two: *t*(6) = 1.167, *p* = .287, Cohen's *d* = 0.48, 5.4% of variance explained.

Finally, we looked at the catch-up saccades themselves. Participants made between 0.2 and 1.2 saccades per transit (600 ms) within our analysis windows (200 ms to 800 ms, and 1200 ms to 1800 ms), with an average of 0.6 saccades per transit for the control and 0.6 saccades per transit for the double-drift condition. At a saccade rate this low, it would not be possible to find the effect of the illusion from saccades alone.

**Figure 5.**
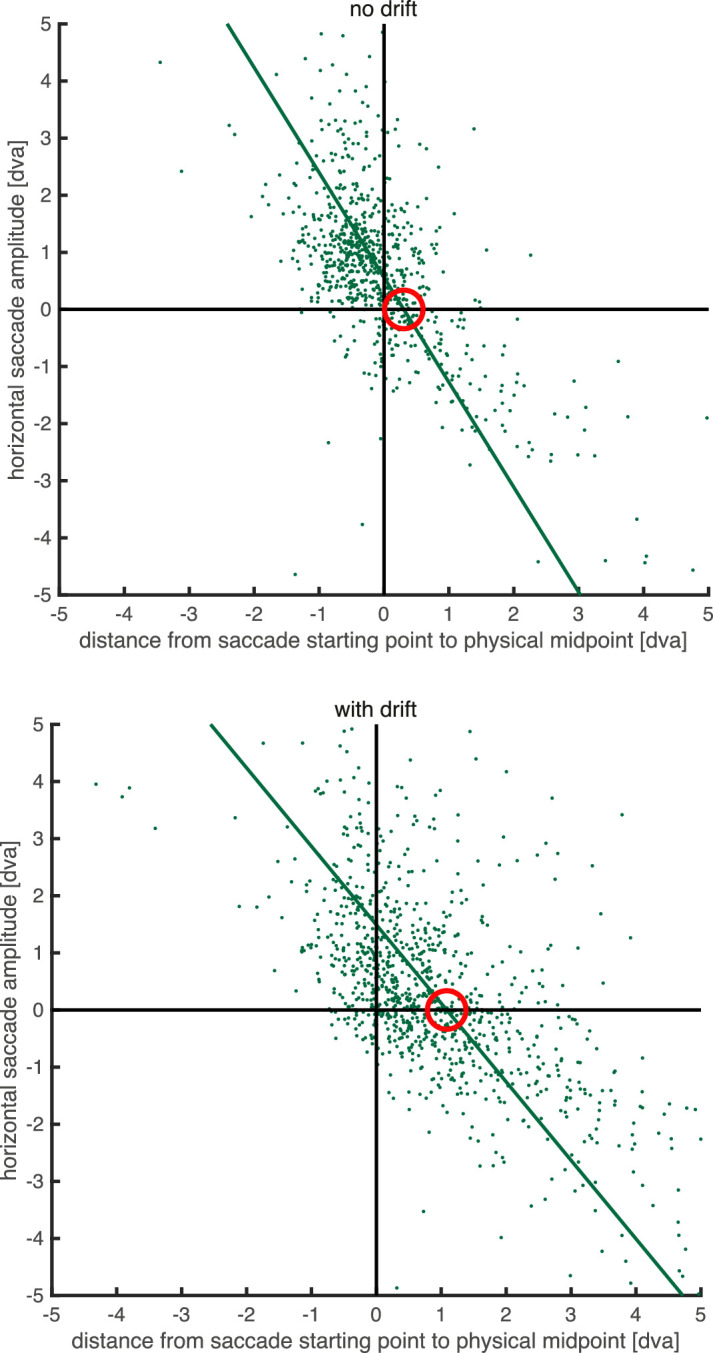
Scatter plot of the *x*-component of saccade vectors. The *x*-axis represents the distance between the saccade starting point and the physical midpoint between the Gabors (positive numbers indicate the direction of illusory motion). The *y*-axis represents the amplitude of the saccade, with positive numbers indicating that the saccade was made in the direction of illusory motion. The green line is the orthogonal regression (Deming, 1943) fit to all saccades across all participants. The *x*-intercept of the green line shows the average *x*-offset between physical midpoint and saccade targets (marked with a red circle), which was the physical midpoint in the control condition, but was offset in the direction of illusory motion in the double-drift condition.

Although the saccades were infrequent, we analyzed their directions to see if they were truly corrective—namely, to determine whether they aimed back to the midpoint of the physical or perceived paths. To this end, we plotted the saccade amplitude as a function of the distance of its starting point from the physical midpoint. We used data based only on the *x*-coordinates (Figure 5) of the saccades occurring within the analysis windows. In other words, we analyzed horizontal saccadic amplitude as a function of horizontal distance from the physical midpoint. For the no-drift conditions, we expect corrective saccades to head to the right (i.e., have a positive amplitude value) if their starting point was left of the physical midpoint (i.e., if the initial distance had a negative value), correcting the retinal offset of the fovea from the virtual target, and vice versa. For the drift conditions, we expect corrective saccades to be to the right if they started to the left of the virtual midpoint of the perceived locations, and vice versa, independent of their location relative to the physical midpoint. The intercept with the *x*-axis of a regression line plotted through these data points reveals the average location of the saccade target, the location where the saccade vector switched from leftward to rightward. As can be seen in Figure 5, the inferred saccade target (*x*-intercept) for the no drift condition was not significantly offset from the physical midpoint; mean offset across participants, 0.31 dva, 95% confidence interval, −0.12 to 0.74 dva, *t*(6) = 1.76, *p* = .129, Cohen's *d* = 0.67, 10.1% of variance explained. In contrast, the *x*-intercept was significantly offset from 0 in the double-drift condition: mean offset across participants, 1.02°, 95% confidence interval, 0.62 to 1.42 dva, *t*(6) = 6.23, *p* < .001, Cohen's *d* = 2.35, 58.1% of variance explained. The *x*-intercept also differed significantly from the *x*-intercept in the no-drift condition: mean difference across participants, 0.71 dva, *t*(6) = 2.66, *p* = .037, Cohen's *d* = 1.01, 20.2% of variance explained. Additionally, we looked at the slopes of these regression lines, because negative slopes would indicate that saccades were corrective. Without drift, the average slope was −2.26 (95%-confidence interval, −3.77 to −0.76), whereas in the drift condition it was −1.29 (95% confidence interval, −1.71 to −0.88). Note that this regression was computed and the *x*-intercept calculated individually for each participant. However, in Figure 5 we plot all saccades from all participants. The inferred saccade target location was consistent with the location of the midpoint between the perceived paths, which suggests that this location rather than the physical midpoint was the target of the catch-up saccades in the double-drift condition. This finding also explains why removing all trials with saccades from the analysis weakened the effect.

## Discussion

In this study, we show that the target for smooth pursuit of the midpoint between two double-drift stimuli is derived from their perceived, not retinal, positions. Corrective saccades likewise seem to be biased in the direction of the perceived rather than actual midpoint. These two findings are in direct contrast with the findings showing that saccades were made to the physical positions of this illusion, when targeting the Gabor directly (Lisi & Cavanagh, 2015; Massendari et al., 2018; Nakayama & Holcombe, 2020). We take these results to mean that the virtual target is calculated from perceived rather than retinal coordinates.

Whether smooth pursuit follows a stimulus or its percept is a question of long standing (Spering & Montagnini, 2011). Here, we report that for paired, double-drift stimuli, the smooth pursuit of their midpoint is driven by perception. First, we replicate earlier findings (Beutter & Stone, 2000; Hafed & Krauzlis 2008; Steinbach, 1976) showing that observers can pursue an inferred location. However, in these earlier studies, the inferred midpoints were based on stimulus components whose perceived locations were aligned with their retinal locations. In our study, the inferred targets are based on stimuli whose perceived locations differ dramatically from their physical locations. In this case, the smooth pursuit clearly followed the midpoint of perceived not physical locations. Thus, although visually guided saccades were found to be driven more by the physical position of the double-drift stimulus (Lisi & Cavanagh, 2015), the smooth pursuit system is instead driven by the perceptual representation of the double-drift stimulus, even while it is present. This outcome is also in line with findings that show smooth pursuit of a motion aftereffect seen on a static stimulus (Braun, Pracejus, & Gegenfurtner, 2006; Matsumiya & Shioiri, 2015). Moreover, the direction of the internal motion of a Gabor (with or against the envelope motion) affected perception and smooth pursuit similarly (Hughes, 2018); however, the perceptual error and smooth pursuit errors were not correlated in this study, suggesting some dissociation between pursuit and perception.

Catch-up saccades were directed to the inferred midpoint between the perceived coordinates of the double-drift rather than its physical coordinates. This result is consistent with past findings that memory-guided saccades to a double-drift stimulus that has vanished also target its last perceived location (Massendari et al. 2018; Ueda, Abekawa, & Gomi, 2018). Thus far, only visually guided saccades to single, visible double-drift stimuli escape the illusion; both memory-guided saccades and saccades to inferred targets are instead largely biased by perceptual processing. This finding also ties in well with other similar studies on saccades targeting the Müller-Lyer illusion (Bruno, Knox, & de Grave, 2010), as well as with the findings from Zivotofsky and colleagues (Zivotofsky et al., 1996; Zivotofsky, Goldberg, & Powell, 2005), showing that targets derived from memory drive saccades to the perceived location of the Duncker illusion.

Although every participant in our study demonstrated the same effect, there were notable individual differences. As can be seen in Figure 3, some of our participants showed an effect akin to a saturation of the illusory effect near the end of the trial. After deviating from the physical direction of the Gabors, their gaze started to follow a line parallel to the physical midpoints but offset in the direction of internal motion by more than one degree. This finding might reflect saturation of the double-drift effect, which has been previously reported (Kwon et al., 2015), and our data would show a variation in this saturation effect across participants with some showing it after 1.5 seconds and some not at all. This finding would suggest individual variation in the way observers weight position and motion information over time. Alternatively, this return of gaze towards the starting point might just reflect anticipatory return to the starting location of the next trial.

In addition to the studies using midpoint tracking (Beutter & Stone, 2000; Hafed & Krauzlis, 2008; Steinbach, 1976), several other investigators have addressed whether smooth pursuit is driven by perception or by the retinal stimulus (for reviews, see Lisberger, 2010; Kowler, 2011; Spering & Montagnini, 2011), but the results have been mixed. Mack, Fendrich, and Wong (1982) reported that, when the retinal and perceived positions of a pursuit target were in conflict, smooth pursuit followed the retinal motion when it was available. Spering and Gegenfurtner (2008) found a dissociation of pursuit speed and perceived speed of motion. In contrast, other authors have found that pursuit is influenced by perception. For instance, with the Duncker illusion, Wyatt and Pola (1979) reported that open-loop smooth pursuit (the first 100 ms after the target's appearance) follows perceived direction even though closed-loop pursuit—after 100ms—does not (Zivotofsky et al., 1995; Zivotofsky, 2005). It should be noted that Zivotofsky's participants pursued the target foveally, which strongly impairs the Duncker illusion, so there might not actually have been a difference between the retinal and perceived directions.

More recently, Ma, Watamaniuk, and Heinen (2017) had participants smoothly pursue a set of four Gabors that had internal motion in addition to translation. They found clear evidence that the internal motion affected both perception and smooth pursuit. The effects were significant but small, for example, about a 2° deviation in pursuit direction when the internal Gabor motion was orthogonal to the translation, compared with the 30° deviation we find in our experiment. This outcome is likely a consequence of the small, 5° separation of their four Gabor patches in their experiment. In this case, gaze direction deviations would be limited to positions within the four Gabors. In addition, the Gabors in their display would fall quite close to the fovea, decreasing the illusion. In our experiment, we use two Gabors separated by 20° and we are able to observe smooth pursuit unconstrained by the stimulus configuration. As a result, we advance these previous findings to show that that an illusion—the double-drift stimulus—influences both perception and closed-loop smooth pursuit equally. We keep the actual stimulus away from the fovea by tracking an inferred midpoint between two double-drift stimuli.

Other studies that have compared the effects of perceived versus retinal motion on optokinetic nystagmus and ocular following have also found mixed results. Logothetis and Schall (1990) found that the slow phase of optokinetic nystagmus followed perception in a binocular rivalry study with monkeys. Spering and Carrasco (2015), using a similar paradigm, found that involuntary eye movements in humans followed the stimulus motion and not perception. Although these involuntary eye movements rely on many of the same cortical processing structures as smooth-pursuit eye movements (Ilg, 1997), our results with smooth pursuit seem to be more consistent with Wyatt and Pola (1979), showing that the open-loop portion of smooth pursuit was driven by perceived direction, as well as the findings by Ma, Watamaniuk, and Heinen (2017), showing that closed-loop smooth pursuit is influenced as well.

Whereas Cavanagh and Tse (2019) stabilized a double-drift stimulus on the retina during smooth pursuit, here we do the opposite; we stabilize the Gabor in perception by having participants smoothly pursue the perceived midpoint between two doubly drifting Gabor patches. This did not stabilize the moving Gabor patches on the retina. When stabilized in perception, the double-drift Gabor patches must move across the retina orthogonally to the pursuit direction. The existence of the illusion here—now measured by the smooth pursuit itself—supports the conclusion that computations underlying the illusion must follow the recovery of the Gabor's motion in world coordinates (i.e., after taking eye movements into account), as well as showing that tracking an inferred target involves using perceived, not retinal, coordinates as input.

There is substantial evidence suggesting that saccades and smooth pursuit are performed by largely overlapping systems (for reviews see Kowler, 2011; Krauzlis, 2004). Why then would smooth pursuit be subject to the double-drift illusion, whereas saccades are not (Lisi & Cavanagh, 2015)? According to Krauzlis (2004), one of the few differences between the circuits underlying smooth pursuit and saccades is the involvement of MT/MST in smooth pursuit. This suggests that MT/MST might be the source of perceptual input to smooth pursuit that is absent in saccades, which is also supported by previous results from smoothly pursuing inferred locations (Beutter & Stone, 2000). The purpose of saccades is not tracking over time, but rapid foveation of peripheral stimuli. Perceived positions emerging in MT/MST or higher (Liu et al, 2019) undoubtedly take more time to compute, explaining one reason why visually guided saccades may be based on direct links from the retina to the superior colliculus, when available (Kato, Takaura, Ikeda, Yoshida, & Isa, 2011), and so avoid the perceptual effects of the double-drift stimulus. We can only speculate whether inferred midpoints between moving stimuli are really computed in MT/MST. This will be a question for future research.

In conclusion, smooth pursuit of inferred targets uses location coordinates that are computed late in the visual position processing hierarchy, after the computation of the double-drift illusion. That is, the tracked target is stabilized in perception, not on the retina. This account of the smooth pursuit system reaffirms the notion that it is not merely controlled by the brainstem or the cerebellum and is instead driven largely by higher visual cortical areas (Krauzlis, 2004) that represent positions in the world as they are consciously experienced.

## Supplementary Material

Supplement 1
